# Two forms of CX3CL1 display differential activity and rescue cognitive deficits in CX3CL1 knockout mice

**DOI:** 10.1186/s12974-020-01828-y

**Published:** 2020-05-14

**Authors:** Aimee N. Winter, Meena S. Subbarayan, Bethany Grimmig, Jason A. Weesner, Lauren Moss, Melinda Peters, Edwin Weeber, Kevin Nash, Paula C. Bickford

**Affiliations:** 1 Center of Excellence for Aging and Brain Repair, Department of Neurosurgery and Brain Repair, USF Morsani College of Medicine, Tampa, FL 33620 USA; 2 Department of Molecular Pharmacology and Physiology, USF Morsani College of Medicine, Tampa, FL 33620 USA; 3 Research Service, James A. Haley Veterans Affairs Hospital, Tampa, FL 33620 USA; 4 Integrated Biomedical Sciences, University of Tennessee Health Science Center, Memphis, TN 38163 USA; 5 Department of Genetics, St. Jude Children’s Research Hospital, Memphis, TN 38105 USA

**Keywords:** Fractalkine, CX3CL1, Neuroinflammation, Microglia, Aging, Neurodegeneration, Cognition, Long-term potentiation, Neurogenesis

## Abstract

**Background:**

Fractalkine (CX3CL1; FKN) is a chemokine expressed by neurons that mediates communication between neurons and microglia. By regulating microglial activity, CX3CL1 can mitigate the damaging effects of chronic microglial inflammation within the brain, a state that plays a major role in aging and neurodegeneration. CX3CL1 is present in two forms, a full-length membrane-bound form and a soluble cleaved form (sFKN), generated by a disintegrin and metalloproteinase (ADAM) 10 or 17. Levels of sFKN decrease with aging, which could lead to enhanced inflammation, deficits in synaptic remodeling, and subsequent declines in cognition. Recently, the idea that these two forms of CX3CL1 may display differential activities within the CNS has garnered increased attention, but remains unresolved.

**Methods:**

Here, we assessed the consequences of CX3CL1 knockout (CX3CL1^-/-^) on cognitive behavior as well as the functional rescue with the two different forms of CX3CL1 in mice. CX3CL1^-/-^ mice were treated with adeno-associated virus (AAV) expressing either green fluorescent protein (GFP), sFKN, or an obligate membrane-bound form of CX3CL1 (mFKN) and then subjected to behavioral testing to assess cognition and motor function. Following behavioral analysis, brains were collected and analyzed for markers of neurogenesis, or prepared for electrophysiology to measure long-term potentiation (LTP) in hippocampal slices.

**Results:**

CX3CL1^−/−^ mice showed significant deficits in cognitive tasks for long-term memory and spatial learning and memory in addition to demonstrating enhanced basal motor performance. These alterations correlated with deficits in both hippocampal neurogenesis and LTP. Treatment of CX3CL1^−/−^ mice with AAV-sFKN partially corrected changes in both cognitive and motor function and restored neurogenesis and LTP to levels similar to wild-type animals. Treatment with AAV-mFKN partially restored spatial learning and memory in CX3CL1^−/−^ mice, but did not rescue long-term memory, or neurogenesis.

**Conclusions:**

These results are the first to demonstrate that CX3CL1 knockout causes significant cognitive deficits that can be rescued by treatment with sFKN and only partially rescued with mFKN. This suggests that treatments that restore signaling of soluble forms of CX3CL1 may be a viable therapeutic option for aging and disease.

## Background

Microglia, myeloid-derived macrophage-like cells, are the resident immune cells of the central nervous system (CNS). Upon detection of an environmental disturbance, microglia become activated and may transition through several activation states in order to eliminate immunogens and/or promote neuroprotection. Indeed, microglia are highly active cells that exist in multiple states, constantly surveying the environment and responding to signals from neurons and other glial cells [1, 2]. As pleiotropic cells, microglia constantly sense and respond differently to their environment depending on the stimuli they encounter. When responding to an insult, microglia release a number of factors that can be inflammatory or cytotoxic such as interleukin (IL)-1β, IL-6, tumor TNF-α, and a number of reactive oxygen and nitrogen species to neutralize immunogens. Although beneficial in the short-term, when prolonged, this form of microglial activation can also promote cellular stress and compromise the health of neural tissue, leading to neuronal damage, neurodegeneration, and subsequent deficits in cognitive or motor function.

To prevent a state of chronic inflammation, microglia are regulated by a number of factors including CD200, CD22, CD47, and fractalkine. Fractalkine (CX3CL1; FKN) is a chemokine that is expressed predominately by neurons in the CNS, with lower levels of expression occurring in astrocytes, and it has been shown to play an important neuroprotective role through the regulation of microglial activity [3, 4]. Specifically, CX3CL1 is known to decrease microglial production of inflammatory mediators by binding to its cognate receptor (CX3CR1) on the surface of microglia (reviewed by [5]). CX3CL1 is constitutively expressed as a membrane-bound protein, which can be cleaved by proteases, such as a disintegrin and metalloproteinase (ADAM) 10 or 17, to generate a diffusible, soluble form of the protein (sFKN [6, 7];). Under normal physiological conditions in the periphery, membrane-bound CX3CL1 has been shown to play a role in the recruiting and adhesion of infiltrating leukocytes [8]. sFKN, on the other hand, acts as both a chemoattractant involved in cellular migration and a neuroprotective signaling molecule that helps maintain microglia in a quiescent state [9, 10]. While membrane-bound CX3CL1 may also bind receptors on the microglial cell surface, recent research suggests that the anti-inflammatory activity of CX3CL1 in the brain is mediated predominately by sFKN [3, 9, 11].

The importance of the CX3CL1/CX3CR1 signaling axis for aging and disease is also well documented. Mice lacking the CX3CR1 receptor display enhanced susceptibility to inflammatory challenge with lipopolysaccharide (LPS) and 1-methyl-4-phenyl-1,2,3,6-tetrahydropyridine (MPTP), chemical models of systemic inflammation, and Parkinson’s disease, respectively [12]. Similarly, CX3CR1 knockout also accelerates disease progression in the G93A mutant Cu, Zn-superoxide dismutase (SOD1) mouse model of amyotrophic lateral sclerosis [12]. We have also shown that mice deficient in CX3CR1 demonstrate several behavioral deficits in both hippocampal- and cerebellar-dependent learning tasks, such as contextual fear conditioning and rotarod, respectively [13]. This effect correlates with increased levels of IL-1β and other inflammatory cytokines and can be blunted by blockade of IL-1β signaling. Furthermore, postnatal CX3CR1^−/−^ mice demonstrate significant abnormalities in synaptic function, such as an increased number of synapses, indicative of impairments in synaptic pruning by microglia, and electrophysiological disturbances consistent with immature synaptic function and impaired development of functional circuits [14]. These alterations in brain connectivity carry into adulthood and correlate with behavioral deficits [15]. Levels of CX3CL1 have been shown to be reduced in aged animals and CSF from Alzheimer’s disease patients [16, 17]. This suggests that perturbations in the CX3CL1/CX3CR1 axis may have a significant impact on cognitive function in aging and disease, and restoring CX3CL1/CX3CR1 signaling may be a viable therapeutic approach to treat these conditions.

Indeed, intrastriatal administration of the chemokine domain of CX3CL1 significantly preserved tyrosine hydroxylase immunoreactivity following injection of 6-hydroxydopamine, and this preservation was accompanied by decreased numbers of activated microglia [18]. Moreover, overexpression of the soluble form of CX3CL1 has been shown to mitigate neurodegeneration induced by overexpression of alpha-synuclein in a model of Parkinson’s disease, and has also been shown to reduce tau phosphorylation and improve cognition in a model of tauopathy [11, 19, 20]. The benefits of CX3CL1 administration have also been observed more generally in aged animals, as treatment with recombinant CX3CL1, comprising only the chemokine domain, significantly increased neurogenesis, and reduced microglial activation in aged rats [16]. The therapeutic potential of CX3CL1 in Alzheimer’s disease is more complex; however, knockout of CX3CR1 appears to be detrimental in mouse models of tauopathy, but beneficial in amyloid expressing mice [21–24].

To date, most studies assessing CX3CL1 activity as it relates to aging and disease have focused predominately on sFKN signaling or signaling of a truncated soluble form containing only the chemokine domain; however, more recent work has begun to take into account the activity of full-length membrane-bound CX3CL1 as well [9, 11, 25]. Emerging evidence suggests that these two forms may display differential effects on aging and neurodegenerative disease processes, and that these differences in activity may be highly context-specific. For example, it has been reported that, similar to CX3CR1 deficiency, amyloid precursor protein/presenilin-1 (APP/PS1) mice lacking CX3CL1 showed reduced amyloid pathology, and expression of obligate sFKN in this context did not improve or exacerbate this effect [25]. Furthermore, deficiency in membrane-bound CX3CL1 specifically resulted in enhanced microglial activation and tau phosphorylation. On the other hand, in models of Parkinson’s disease, it was shown that membrane-bound CX3CL1 had no effect on overall disease progression and pathology while sFKN administration significantly improved motor function and preserved TH-positive neurons in the *substantia nigra* [9, 11]. These studies suggest that membrane-bound CX3CL1 and sFKN may display differing degrees of therapeutic efficacy depending on disease context; however, the individual roles of membrane-bound CX3CL1 and sFKN on motor function and cognition in a normal physiological setting have not yet been elucidated and may shed light on the therapeutic benefits and functions of each form of CX3CL1.

In this study, we confirm that CX3CL1 deficiency was sufficient to induce cognitive impairment. Furthermore, we used CX3CL1 knockout (CX3CL1^−/−^) mice to evaluate the differential abilities of both a mutated, obligate membrane-bound form of CX3CL1 (mFKN) and sFKN to rescue deficits caused by suppressed CX3CL1 signaling. To our knowledge, our results are the first to demonstrate that loss of CX3CL1 leads to significant cognitive impairment, in good agreement with our previous observations for CX3CR1, and to define the differing activities of mFKN and sFKN in this context.

## Methods

### AAV Production

Recombinant AAV serotype PhP.B (rAAV) vectors expressing either mFKN or sFKN (GI 114431260) were cloned using PCR on cDNA isolated from mouse brain as previously described [9]. sFKN protein expressed using this vector comprises amino acids 1-336, which includes both the chemokine domain and mucin-like stalk. mFKN DNA contained two mutations (R337A and R338A) to prevent cleavage by ADAM10/17 into the soluble form. mFKN protein expressed using this vector comprises all 395 amino acids of the full-length CX3CL1 protein with arginine to alanine substitutions at positions 337 and 338. mFKN DNA contained two mutations (R337A and R338A) to prevent cleavage by ADAM10/17 into the soluble form. The vector includes the AAV2 terminal repeats and chicken beta-actin (CBA) promoter for mRNA transcription of mFKN and sFKN. Both sFKN and mFKN were tagged with hemaglutinin (HA) at the C-terminus for easy detection of exogenous protein. rAAV particles were quantified using a modified dot plot protocol as described by Burger and Nash [26] and are expressed as vector genomes (vg)/mL.

### Animals

The following work using animals was conducted according to the NIH guidelines for animal use and IACUC of the University of South Florida College of Medicine. CX3CL1^−/−^ mice (Merck Sharp and Dohme Corp.) were obtained with a material transfer agreement and maintained in a colony with WT littermates at the University of South Florida. Genotyping was outsourced and performed using a commercially available service (Transnetyx Inc. Cardova, TN). Only male mice were used for experiments. Mice were treated at 2 months of age with a single tail vein injection of rAAV expressing either green fluorescent protein (GFP), mFKN, or sFKN at a concentration of 7x10^12^ vg/mL, followed by behavioral assessment between 3 and 4 months of age. Animals were maintained on a 12-h light/dark cycle and ad libitum access to food and water.

### Behavioral testing

#### Open Field

General locomotion and exploratory activity were assessed by observing the animals in a novel environment. The mice were placed in 40 cm square box and allowed to navigate the space for 15 min. Distance traveled around the arena was recorded and quantified with ANY-Maze software from Stoelting.

#### Rotarod

Mice were tested on the rotarod apparatus (UgoBasile) in order to examine innate motor coordination and motor learning and performance over time. This machine consists of a 3-cm diameter textured rod, initially rotating at 4 rpm when the mice begin testing, and accelerating to 40 rpm within 5 min. Trials were terminated once the mouse fell off the beam onto a platform, denoting the latency to fall. The mice completed 4 trials a day with 30-min intertrial interval, over 2 days for a total of 8 trials.

#### Fear conditioning

Mice were placed in a 17 × 17-cm plexiglass box within a soundproofed chamber and allowed to acclimate to the novel environment for 3 min, while locomotion and baseline freezing behavior were evaluated by the ANY-Maze software. Freezing behavior in the program was operationally defined as 2 or more consecutive seconds of motionless inactivity and was corroborated by hand scored data. White noise and a gentle fan were used inside the chamber to reduce attention to noise cues that may be present outside of the testing room. A 30-s 90-dB tone (cued stimulus) was delivered to the apparatus at 3 min followed by a foot shock (0.5 mA), which was administered during the last 2 s of the tone, so that the two stimuli terminate simultaneously. This stimulus block was repeated at 5 min, and freezing behavior was assessed until the 7th min of the test.

Mice were then re-exposed to the context at 24 h and 2 weeks post-training in order to observe the freezing behavior after training. Association of the context with an aversive stimulus is mediated by the hippocampus, and freezing behavior is representative of hippocampal function. Mice were placed back into the conditioning chamber with all of the cues present during training, but received no foot shock. Freezing behavior was monitored and analyzed at 14 days post training.

Mice were also tested in an altered context test in which all salient cues are removed, except for the tone. Freezing behavior was observed in a novel context with and without the tone to determine the association to the tone and shock. This association is mediated by the amygdala.

#### Barnes maze

Barnes maze was conducted after 6 pm during the animals’ dark cycle and under bright lights to encourage mobility throughout the testing. Moreover, a continuous 2500-Hz tone was played throughout both training and testing phases to further encourage mobility and escape. Behavior in the arena was recorded and analyzed using the ANY-Maze software. Training is consisted of 4 trials daily for 4 days. Each trial was terminated when the mouse completely escaped through the target hole or after 3 min had elapsed. After each trial, the mouse was allowed to remain in the covered target hole for 30 s, and the tone was silenced upon entry into the escape chamber. A probe trial was tested at 24 h after training, by replacing the escape chamber with a false bottom to prevent entry. Recall was assessed by comparing the number of entries into each hole around the maze.

### Electrophysiology

Following behavior experiments, a cohort of mice (*n* = 4/group) was euthanized, and the hippocampus was dissected out for field potential LTP recordings as previously described [27, 28]. The brain was removed rapidly and placed in oxygenated ice cold cutting solution with the following composition: 110 mM sucrose, 60 mM NaCl, 3 mM KCl, 1.25 mM NaH_2_PO_4_, 28 mM NaHCO_3_, 5 mM glucose, 0.6 mM ascorbate, 0.5 mM CaCl_2_, and 7 mM MgCl_2_. Hippocampal slices were cut on a vibratome to 400 μm thickness filled with ice cold cutting solution. The slices were then incubated with 50% cutting solution and 50% artificial cerebrospinal fluid solution (ACSF) with the following composition: 125 mM NaCl, 2.5 mM KCl, 1.25 mM NaH_2_PO_4_, 25 mM NaHCO_3_, 25 mM glucose, 2 mM CaCl_2_, and 1 mM MgCl_2_ with constant 95% O_2_/5% CO_2_ for 10 min to equilibrate. Slices were then transferred onto the nylon mesh of the recording chamber (Automate Scientific) and allowed to recover for 1 h with constant supply of oxygenated ACSF (rate 1 ml/min). The recording chamber was maintained at 30 °C ± 0.5 °C. Following recovery, the Schaffer collaterals rising from CA3 region of the hippocampus were stimulated using electrodes made of formvar-coated nichrome wire that delivers biphasic stimulus pulses (1–15 V, 100 μs duration, 0.05 Hz). The delivery of the stimulation was controlled by the pClamp 9.0 software (Molecular Devices) using a stimulus isolator (model 2200; A-M systems) and Digidata1322 A interface (Molecular Devices). The field excitatory post-synaptic potential (fEPSPs) recordings were recorded from the stratum radiatum in CA1 region of the hippocampus using glass electrodes filled with ACSF with resistance 1–4 mΩ. The signals obtained were amplified using a differential amplifier (model 1800; A-M systems). The amplified signals were filtered at 1 kHz and digitized at 10 kHz. The input output curve was determined by stimulating the slices in 0.5 mV increment from 0 to 15 mV. The 50% of the maximum fEPSPs determined using the input output curve was used as the baseline stimulus intensity in all the experiments. Paired-pulse facilitation (PPF) was performed to measure short-term plasticity. The slices were stimulated at 50% of the maximum intensity with sequential pulses for every 2 s up to 30 s. For LTP field potential recordings, the slices were stimulated with theta burst protocol containing two trains of four pulse bursts at 100 Hz separated by 20 s, repeated six times with an intertrain interval of 10s. For analysis, the last 20 min of the fEPSP recording was averaged and compared.

### Tissue isolation

Mice received lethal intraperitoneal injections of sodium pentobarbital. Once deeply anesthetized, they were transcardially perfused with 0.1 M PBS. The brains were removed then cut in half along the midline. One hemisphere was allowed to chill in ice cold PBS before rapidly dissecting out areas of interest, which were flash frozen in liquid nitrogen. The other hemisphere was drop fixed in 4% paraformaldehyde for 24 h, which was replaced with a 30% sucrose solution for cryoprotection.

### ELISA quantification of CX3CL1

Flash frozen brain tissue, excluding the hippocampus and cortex, was homogenized using an electric tissue homogenizer in 1:10 weight-to-volume ratio of ice-cold RIPA buffer (Millipore) containing protease inhibitors and EDTA (Pierce). Lysates were then centrifuged at 10,000×*g* at 4 °C for 15 min, and supernatant was collected and analyzed to determine protein concentration using a BCA assay (Pierce). Lysates were then analyzed using ELISA kit with antibodies directed towards the N-terminus of the CX3CL1 protein, such that all forms of the CX3CL1 protein were detected. Samples were loaded in triplicate at a concentration of 50 μg total protein per well and incubated overnight at 4 °C. Following incubation, the manufacturer’s (R&D Systems) suggested protocol was followed. Optical density values for each ELISA plate were measured on a plate reader (BioTek), and sample concentrations for total CX3CL1 were calculated based upon the supplied standard curve.

### Immunohistochemistry

Immunohistochemical analysis was conducted using 40 μm thick sections for every 6th sagittal section of the right hemisphere. In order to ensure sampling of the entire hippocampus, the sections collected included those immediately medial and lateral of the hippocampus. Free floating sections were treated with 3% hydrogen peroxide in methanol to remove endogenous peroxidase activity. They were blocked with 10% normal serum corresponding to the species each secondary antibody was raised in (horse or goat) with 0.1% Triton X-100 diluted in PBS. Primary antibodies to doublecortin (DCX; Santa Cruz, SC-8066; 1:150) or Ki67 (Novocastra, NCL#Ki67p; 1:500) were also diluted with 3% serum with 0.1% Trition X-100 overnight, oscillating at 60 rpm at 4°. Secondary biotinylated antibodies were diluted 1:500 and 1:1000 in PBS containing 3% normal serum with 0.1% Triton X-100 for DCX and Ki67, respectively, and incubated for 2 h at room temperature. Avidin-biotin complex was used to amplify signal (Vector Labs), and diaminobenzadine (Sigma) was used for color development.

### Stereology

Cells labeled positively for either DCX or Ki67 were quantified in the subgranular zone (SGZ) of the dentate gyrus (DG) on a Nikon Eclipse 600 microscope using the optical fractionator method of unbiased stereology and Stereo Investigator software (MicroBrightField). A grid size of 225 × 225 and counting frame of 150X150 was employed for both for DCX and 100 × 100 grid size and counting frame for Ki67 staining such that at least 200 cells were counted for each animal. Anatomical structures were outlined using a 10×/0.45 objective, and cells were counted using a 40 × 0.95 objective.

## Results

### CX3CL1 is expressed in brain tissue following tail vein injection of AAV

**Fig. 1:**
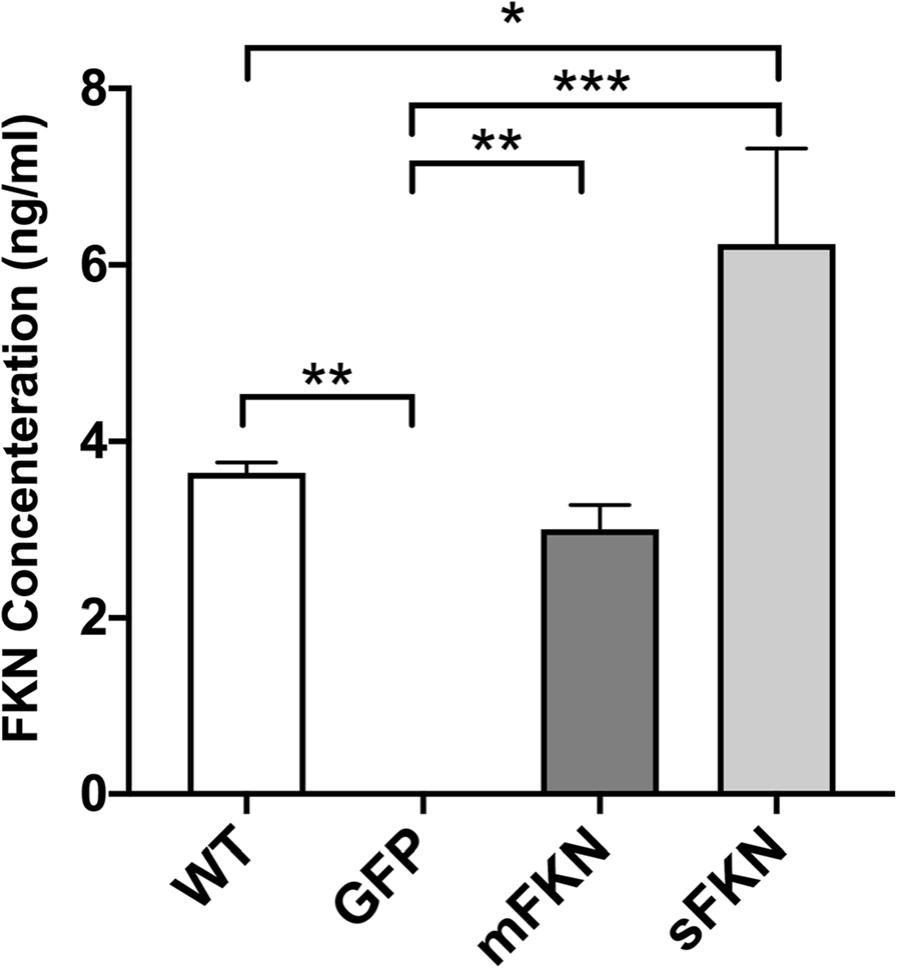
FKN is expressed in brain tissue following tail vein injection of adeno-associated viral vector (AAV). Quantitative assessment of FKN expression in brain homogenates. Animals were sacrificed, and brain tissue was collected for analysis by ELISA. CX3CL1^−/−^ mice treated with GFP displayed negligible levels of FKN expression in brain while mice injected with AAV expressing mFKN displayed expression levels similar to that of WT mice. Animals receiving AAV expressing sFKN demonstrated high levels of expression in comparison to WT controls. Data were analyzed by one-way ANOVA (*n* = 4, *F* (3, 13) = 22.85; *p* < 0.0001) with Tukey’s post hoc test. *p < 0.05, **p < 0.01, and ***p < 0.001

Approximately 2 months following tail vein injection of the rAAV vectors expressing GFP or CX3CL1, brain tissue was isolated and homogenized to assess expression of CX3CL1. Expression of exogenous sFKN or mFKN in the brain was evaluated using ELISA. Expression was observed only in CX3CL1^−/−^ animals that received rAAV expressing sFKN, mFKN, or in WT animals, while CX3CL1^−/−^ mice that received rAAV expressing GFP did not display any positive immunoreactivity (Fig. 1). Mice receiving AAV-mFKN demonstrated expression at levels similar to those observed in WT mice expressing endogenous CX3CL1, while mice that received the vector expressing sFKN displayed levels that were approximately twice that of WT controls.

### CX3CL1^−/−^ mice demonstrate impaired associative learning and long-term memory that is disparately impacted by mFKN and sFKN

**Fig. 2:**
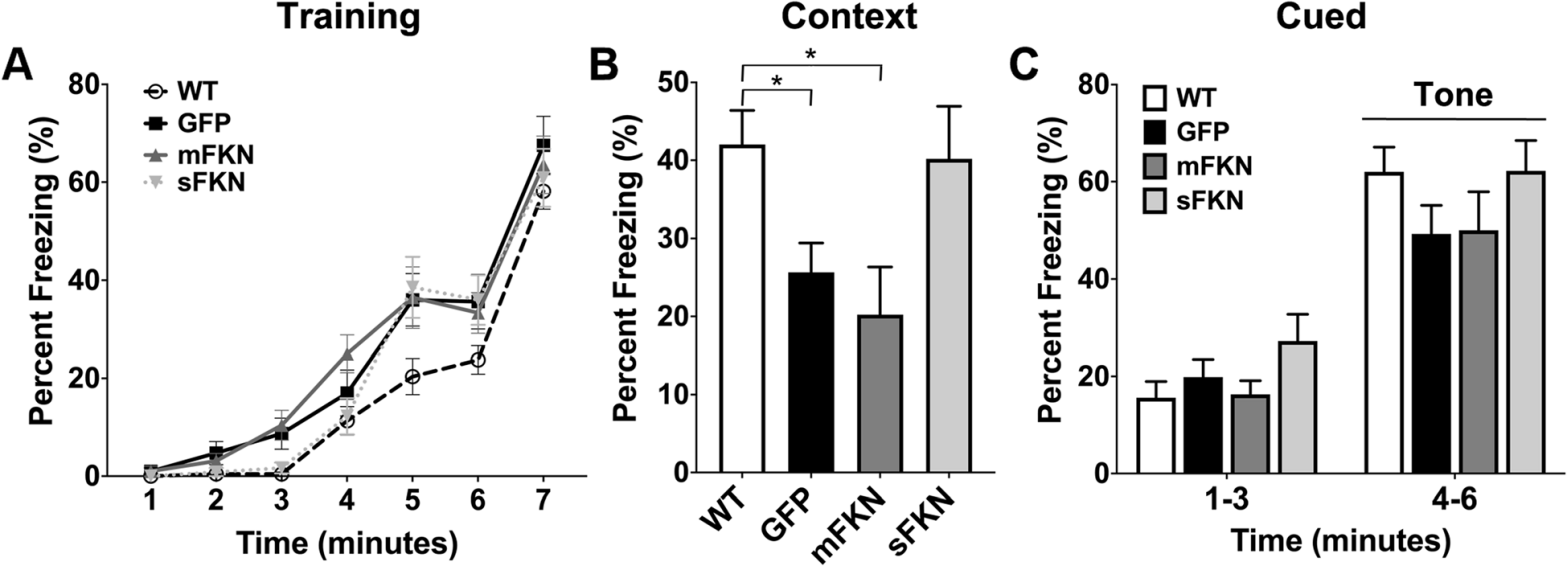
CX3CL1^−/−^ mice demonstrate impaired associative learning and long-term memory that is disparately impacted by mFKN and sFKN. **a** Mice were subjected to a standard two-shock training protocol for fear conditioning. Shocks were administered at 3 min and 5 min, and freezing behavior was assessed before and after each shock for a duration of 7 min. All groups of mice performed similarly in this training paradigm. Data were analyzed using two-way ANOVA for repeated measures (*n* = 8, *F* (18, 288) = 1.486, *p* = 0.09). **b** After 2 weeks, mice were placed back into the context in which they initially experienced a foot shock and monitored for freezing behavior over the course of 3 min. CX3CL1^−/−^ mice that received AAV-GFP showed significantly less freezing behavior than their WT counterparts. Mice that were administered the AAV-mFKN also showed a similar trend in decreased freezing behavior. Mice administered AAV-sFKN showed a trend towards increased freezing behavior that was more similar to WT mice than mice treated with GFP; however, this trend did not reach significance. Data were analyzed by one-way ANOVA (*n* = 8, *F* (3, 65) = 4.210; *p* < 0.010) with Tukey’s test. *p ≤ 0.05. **c** Mice were allowed 3 min to acclimate in a novel context before presentation of the conditioned stimulus (tone) for three additional minutes. Freezing was monitored for the duration of the test. All mice displayed normal freezing behavior in response to the conditioned stimulus, and no significant differences were observed between groups. Data were analyzed by two-way ANOVA for repeated measures (*n* = 8, *F* (3, 44) = 1.132; *p* = 0.347)

Contextual and cued fear conditioning was used to assess associative learning and long-term memory in CX3CL1^−/−^ mice. Mice were subjected to a standard two-shock training protocol as described above, and freezing behavior was assessed before and after each shock for a duration of 7 min. All groups of mice performed similarly in this training paradigm (Fig. 2a). As we determined that no differences in contextual memory between WT mice and CX3CL1^−/−^ mice were present after 24 h (data not shown), mice were allowed 2 weeks to rest during which no testing took place. After 2 weeks, mice were placed back into the context in which they initially experienced a foot shock and monitored for freezing behavior over the course of 3 min. At this later time point, CX3CL1^−/−^ mice that received GFP showed significantly less freezing behavior than their WT counterparts (Fig. 2b). Mice that were administered the AAV-mFKN also showed a similar decrease in freezing behavior compared to WT controls. On the other hand, mice administered AAV-sFKN showed increased freezing behavior that was not significantly different from WT mice, but did not reach significance versus CX3CL1^−/−^ mice treated with GFP (Fig. 2b).

Freezing in response to a conditioned stimulus in a novel context was also observed after 2 weeks. Mice were allowed 3 min to acclimate to the novel context before presentation of the conditioned stimulus (tone) for three additional minutes. All mice displayed normal freezing behavior in response to the conditioned stimulus, and no significant differences were observed between groups (Fig. 2c).

### CX3CL1^−/−^ mice display impairments in the Barnes maze test for spatial memory that are corrected by treatment with mFKN and sFKN

**Fig. 3:**
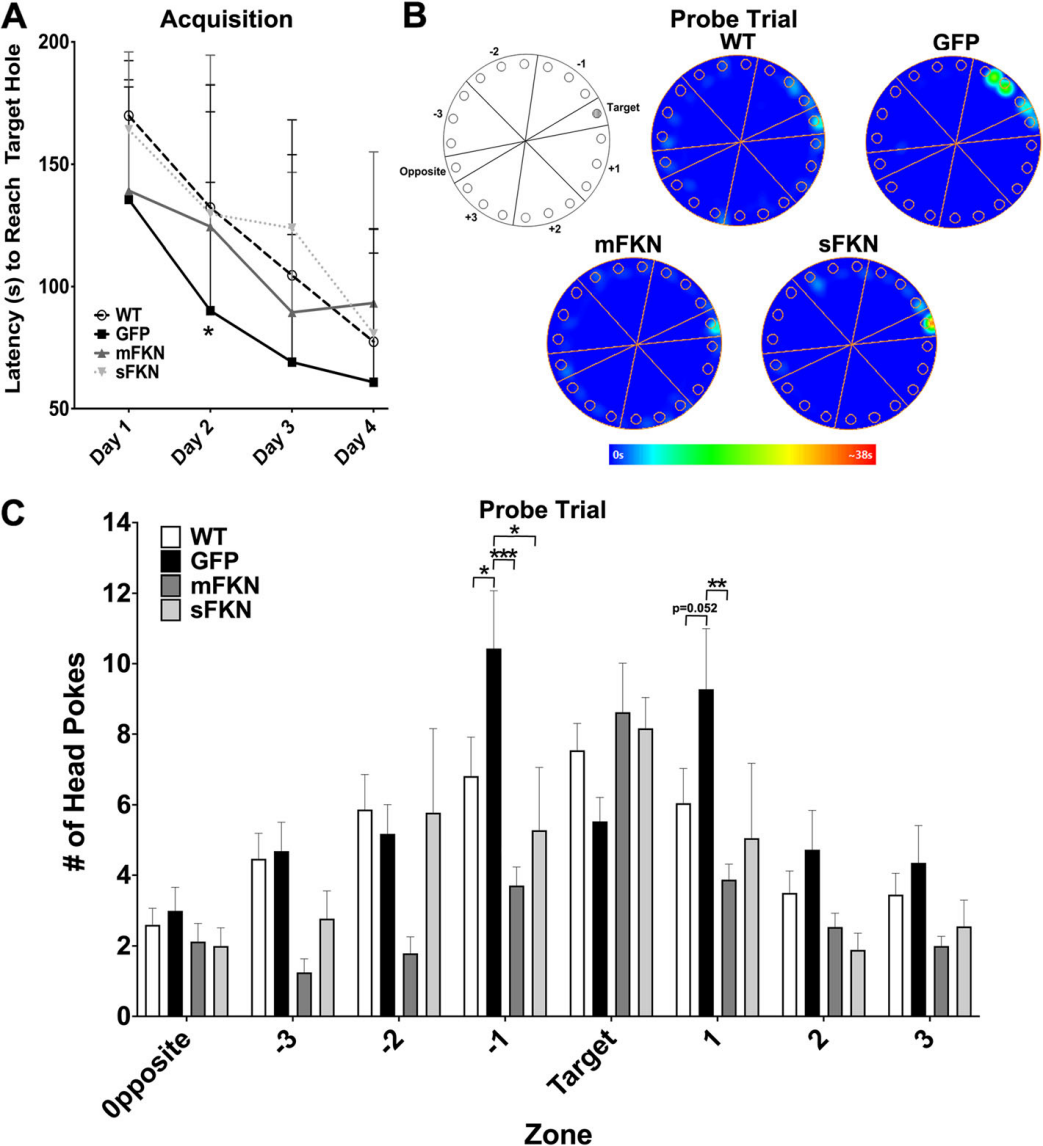
CX3CL1^−/−^ mice display impairments in the Barnes maze test for spatial memory that are corrected by treatment with mFKN and sFKN. **a** Mice were assessed for spatial learning and memory using a Barnes maze task. Mice were subjected to 4 days of training during which they were taught to locate an escape pod beneath one of the holes on the perimeter of the maze. All mice learned the task at a similar rate regardless of treatment of genotype. Data were analyzed by two-way ANOVA for repeated measures (*n* = 8, *F* (9, 153) = 1.876, *p* = 0.059). **b** On the fifth day, the escape pod was replaced by a false bottom, and mice were allowed to explore the maze for 1 min. The figure shows a map of the Barnes maze zones and representative heat maps for each treatment group. The maze was split into eight zones for scoring purposes as indicated. Representative heatmaps of one animal from each treatment group were generated to illustrate the different exploration strategies observed for each group during the probe test, and indicate the relative amount of time spent at different locations around the maze. **c** Quantitative assessment of the number of times each mouse poked its head into each hole of the Barnes maze. WT mice explored the target hole more often than any other hole in the maze, with the number of head pokes decreasing as distance from the target hole increased. mFKN- and sFKN-treated animals displayed a similar trend. CX3CL1^−/−^ mice that were administered GFP displayed a trend towards decreased exploration of the target hole compared to WT animals, although this difference did not reach significance. Mice that received AAV expressing GFP explored the three adjacent holes on either side of the target (zones ^−^ 1 and + 1) the most and made significantly more head pokes in the − 1 zone than their WT counterparts. Mice that were treated with either mFKN or sFKN showed a trend towards increased exploration of the target zone, similar to WT mice, and showed significantly less exploration in the − 1 (mFKN and sFKN) and + 1 (mFKN) zones than mice receiving GFP. Data were analyzed using two-way ANOVA for repeated measures (*n* = 8, *F* (21, 329) = 2.482, *p* < 0.001) with Tukey’s test. *p < 0.05, **p < 0.01, and ***p < 0.001 for each respective zone

Cognition was also assessed by evaluating spatial learning and memory in WT and CX3CL1^−/−^ mice using a Barnes maze task. Mice were subjected to 4 days of training during which they were taught to locate an escape pod beneath one of the holes on the perimeter of the maze (Fig. 3b). All mice learned the task at a similar rate regardless of treatment or genotype (Fig. 3a).

On the fifth day, the escape pod was replaced by a false bottom, and mice were allowed to explore the maze for 1 min. The number of times each mouse poked its head into each hole was then observed. WT mice explored the target hole more often than any other hole in the maze, with the number of head pokes decreasing as distance from the target hole increased. mFKN- and sFKN-treated animals displayed a similar trend (Fig. 3b, c). CX3CL1^−/−^ mice that were administered GFP displayed a trend towards decreased exploration of the target hole compared to WT animals, although this difference did not reach significance (Fig. 3c). Mice that received AAV-GFP explored the three adjacent holes on either side of the target (zones − 1 and + 1) the most and made significantly more head pokes in the − 1 zone than their WT counterparts. Mice that were treated with either mFKN or sFKN showed a trend towards increased exploration of the target zone, similar to WT mice, and showed significantly less exploration in the − 1 (mFKN and sFKN) and + 1 (mFKN) zones than mice receiving GFP (Fig. 3b, c).

### CX3CL1^−/−^ mice display enhanced motor performance that is differentially affected by mFKN and sFKN

**Fig. 4:**
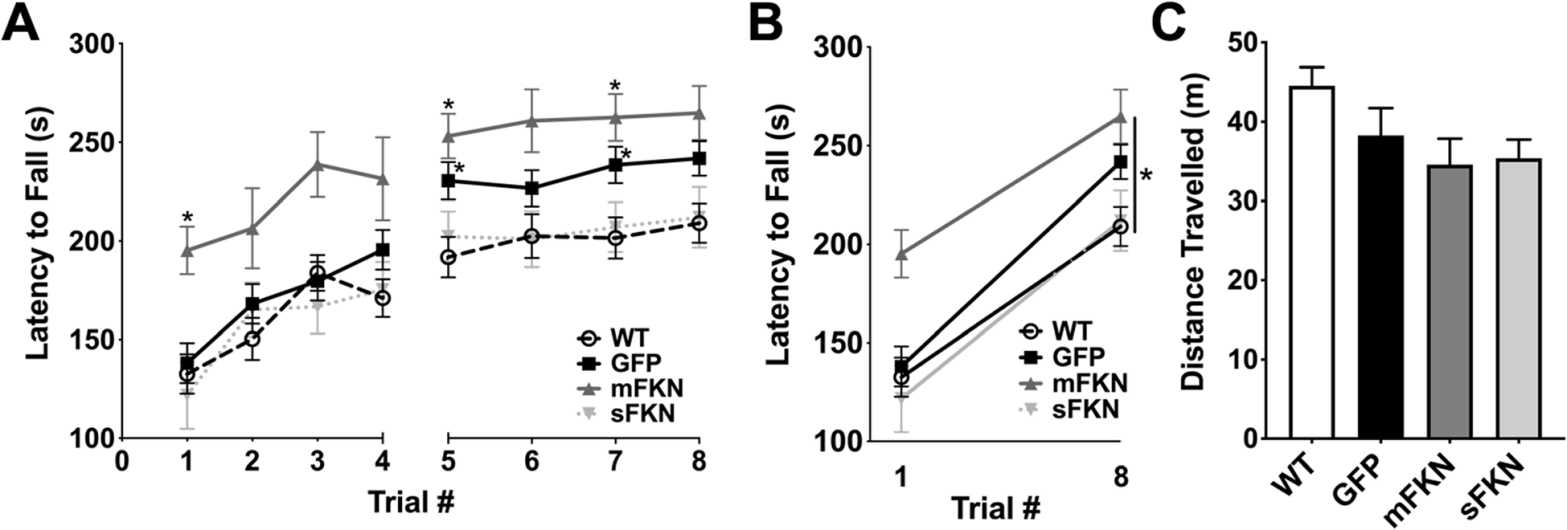
CX3CL1^−/−^ mice display increased baseline motor performance that is differentially affected by mFKN and sFKN. **a** Mice were assessed by accelerating rotarod over a period of 2 days. On day 1 (trials 1–4), no difference in motor performance was observed between WT mice, and CX3CL1^−/−^ mice administered a AAV expressing either GFP or sFKN; however, CX3CL1^−/−^ mice treated with AAV expressing mFKN demonstrated superior motor performance in comparison to WT mice (trials 1–4) and GFP mice (trial 1). On day two of testing (trials 5–8), mice treated with GFP displayed significantly enhanced motor performance in comparison to WT controls (trials 5 and 7). Treatment with mFKN and sFKN differentially impacted motor coordination with mFKN significantly enhancing motor performance in comparison to WT mice (trials 5–8), and showing a trend towards enhanced coordination compared to CX3CL1^−/−^ mice treated with GFP. On the other hand, CX3CL1^−/−^ mice treated with sFKN displayed a trend towards decreased motor coordination in comparison mice treated with GFP, and performed more similarly to WT controls. Data were analyzed using two-way ANOVA for repeated measures (*n* = 8, *F* (3, 101) = 4.826, *p* < 0.01) with Tukey’s test to compare differences between groups. *p < 0.05 and **p < 0.01 in comparison to WT controls for each trial. **b** All mice showed significant improvement in motor coordination between trials 1 and 8. Data were analyzed by two-way ANOVA for repeated measures (*n* = 8, *F* (1, 101) = 84.61, *p* < 0.0001) with Sidak’s test to compare differences between trials within each treatment group. *p < 0.05 in comparison to trial 1. Slopes are not significantly different, as determined by linear regression (*F* (3, 202) = 0.8176, *p* = 0.485), suggesting that all mice learned the task at a similar pace. **c** Mice were observed using an open field paradigm to assess spontaneous locomotion. No differences in total distance travelled were observed between any of the treatment groups. Data were analyzed by one-way ANOVA (*n* = 8, *F* (3, 57) = 2.632, *p* = 0.0586)

To determine if CX3CL1 knockout or treatment with sFKN or mFKN affected motor function, mice were assessed by accelerating rotarod over a period of 2 days. On day 1 (trials 1–4), no difference in motor performance was observed between WT mice, and CX3CL1^−/−^ mice administered AAV expressing either GFP or sFKN; however, CX3CL1^−/−^ mice treated with AAV expressing mFKN demonstrated increased latency to fall in comparison to WT mice (trials 1–4) and GFP mice (trial 1). On day two of testing (trials 5–8), CX3CL1^−/−^ mice treated with GFP displayed significantly longer latency to fall in comparison to WT controls (trials 5 and 7). Treatment with mFKN and sFKN differentially impacted motor coordination, with mFKN significantly increasing latency to fall in comparison to WT mice (trials 5 and 7), and showing a trend towards enhanced coordination compared to CX3CL1^−/−^ mice treated with GFP. On the other hand, CX3CL1^−/−^ mice treated with sFKN performed similarly to WT controls (Fig. 4a). Latency to fall was then compared for trials 1 and 8 to assess overall improvement in motor coordination and indicative of motor learning. All mice showed significant improvement in motor coordination and appeared to learn the task at similar rates despite increased baseline motor coordination in mFKN-treated animals (Fig. 4b).

Mice were also subjected to an open field test to determine if CX3CL1 knockout impacted spontaneous locomotion, which may in turn impact rotarod performance and fear conditioning. All mice showed similar levels of exploration in the open field arena as measured by distance traveled, suggesting that neither CX3CL1 knockout nor treatment with sFKN or mFKN affects spontaneous activity (Fig. 4c).

### CX3CL1^−/−^ mice show deficits in LTP that are mitigated by treatment with mFKN and sFKN

**Fig. 5:**
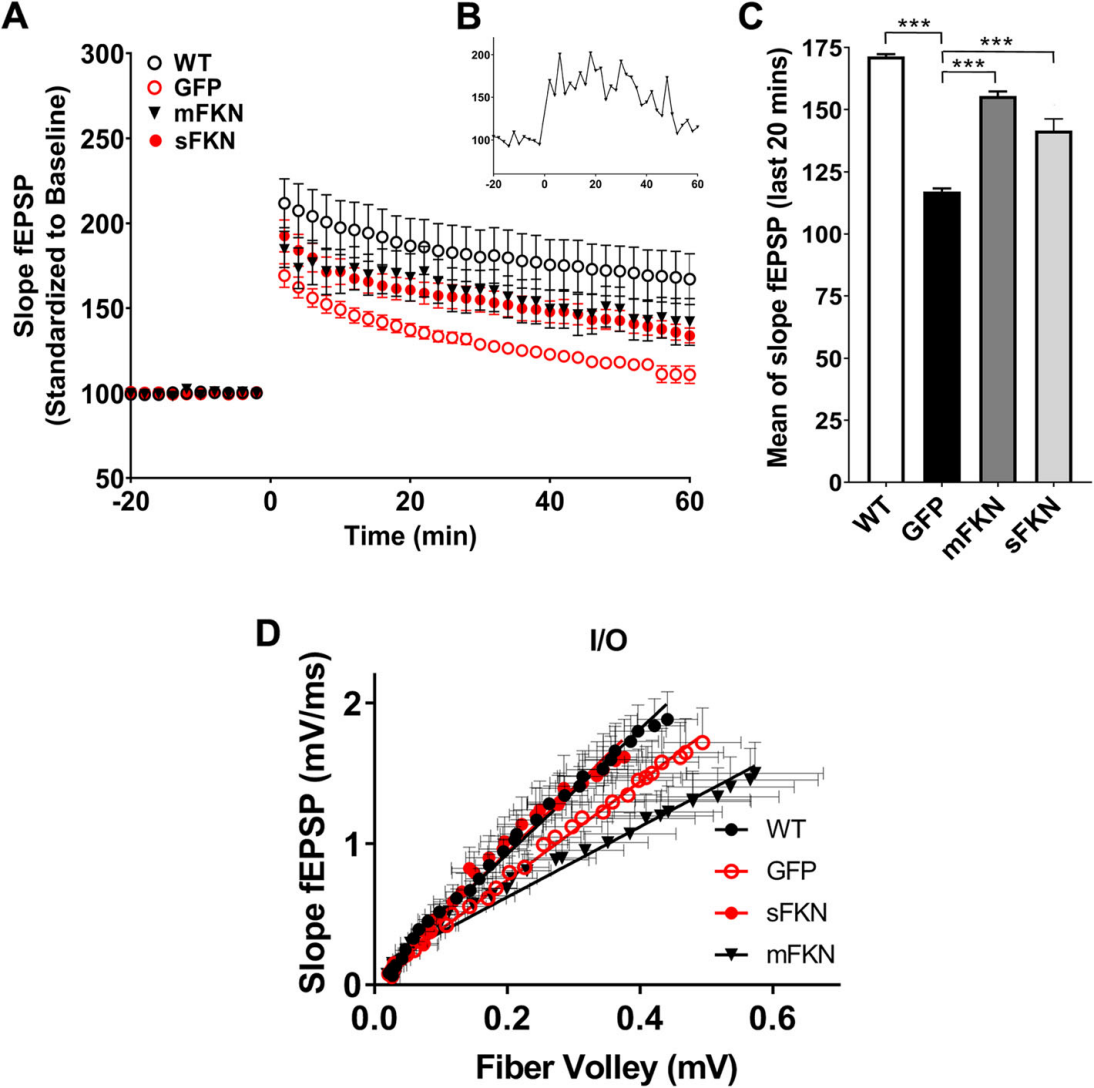
CX3CL1^-/-^ mice show deficits in LTP that are mitigated by treatment with mFKN and sFKN. **a** LTP was induced in hippocampal slices by high frequency stimulation (2 train, 4 Pulse, 1 s, 100 Hz bursts separated by 20 s with intertrain interval of 10s) following baseline recordings conducted over a period of 20 min. Changes in fEPSP slope were then monitored for a duration of 60 min and expressed as a percent of baseline. In comparison to WT controls, CX3CL1^−/−^ animals treated with AAV expressing GFP demonstrated significantly impaired potentiation that decayed over time to baseline levels. In contrast, treatment with either mFKN or sFKN partially preserved LTP in comparison to mice treated with GFP (*n* = 14). **b** Representative single signal of CX3CL1^−/−^ mice treated with mFKN showing instability in maintaining the LTP post theta burst stimulation. **c** Mean slope of the fEPSP was calculated for the last 20 min of the monitoring period, confirming that CX3CL1^−/−^ mice treated with GFP show significant deficits in LTP that are partially, but significantly ameliorated by treatment with either mFKN or sFKN. Data were analyzed by one-way ANOVA (*n* = 11, *F* (1.5, 15.03) = 1696, *p* < 0.0001) with Tukey’s test. ***p < 0.001. **d** The input/output curve for CX3CL1^−/−^ mice treated with sFKN were closer together with WT mice, whereas CX3CL1^−/−^ mice treated with mFKN were closer together with CX3CL1^−/−^ mice treated with GFP. Slopes are significantly different as determined by linear regression (*n* = 30, *F* (3, 112) = 170, *p* < 0.0001) suggesting that the presynaptic input and the post synaptic outpour for CX3CL1^−/−^ mice treated with GFP and mFKN were different from that of WT and CX3CL1^−/−^ mice treated with sFKN

We have previously shown that CX3CR1^−/−^ mice show cognitive dysfunction in hippocampal-dependent tasks that correlates with decreased LTP [13]. Given the similar cognitive deficiencies observed in CX3CL1^−/−^ mice in the current study, we next evaluated LTP to determine if these mice display altered synaptic plasticity. LTP was induced in hippocampal slices by theta burst stimulation (five, four pulse, 200 Hz bursts separated by 20 s) following baseline recordings conducted over a period of 20 min. Changes in fEPSP slope were then monitored for a duration of 60 min and expressed as a percent of baseline. In comparison to WT controls, CX3CL1^−/−^ animals treated with AAV-GFP showed impaired LTP and returned to baseline levels as shown in the analysis of the last 20 min (Fig. 5c). In contrast, stimulation of the Schaffer collateral resulted in strong LTP response in slices from the sFKN-treated CX3CL1^−/−^ mice similar to that observed for WT animals (Fig. 5a, c). Though the slices from the mFKN-treated CX3CL1^−/−^ mice showed robust LTP maintenance on average, the individual fESP signals recorded from the stratum radiatum of CA3 were quite variable as noted in the large error bars and shown in Fig. 5b, suggesting that sFKN and mFKN may not have equivalent actions. The efficacy of the neurotransmitter release measured using an input-output curve was closer for sFKN-treated CX3CL1^−/−^ and WT mice, whereas the mFKN treated CX3CL1^−/−^ mice were similar to GFP-injected CX3CL1^−/−^ mice. These data indicate that CX3CL1^−/−^ mice showed significant impairment in hippocampal plasticity and treatment with both sFKN and mFKN rescued the impairment, although to different extents.

### CX3CL1^−/−^ mice show deficits in neurogenesis that are rescued by treatment with sFKN, but not mFKN

**Fig. 6:**
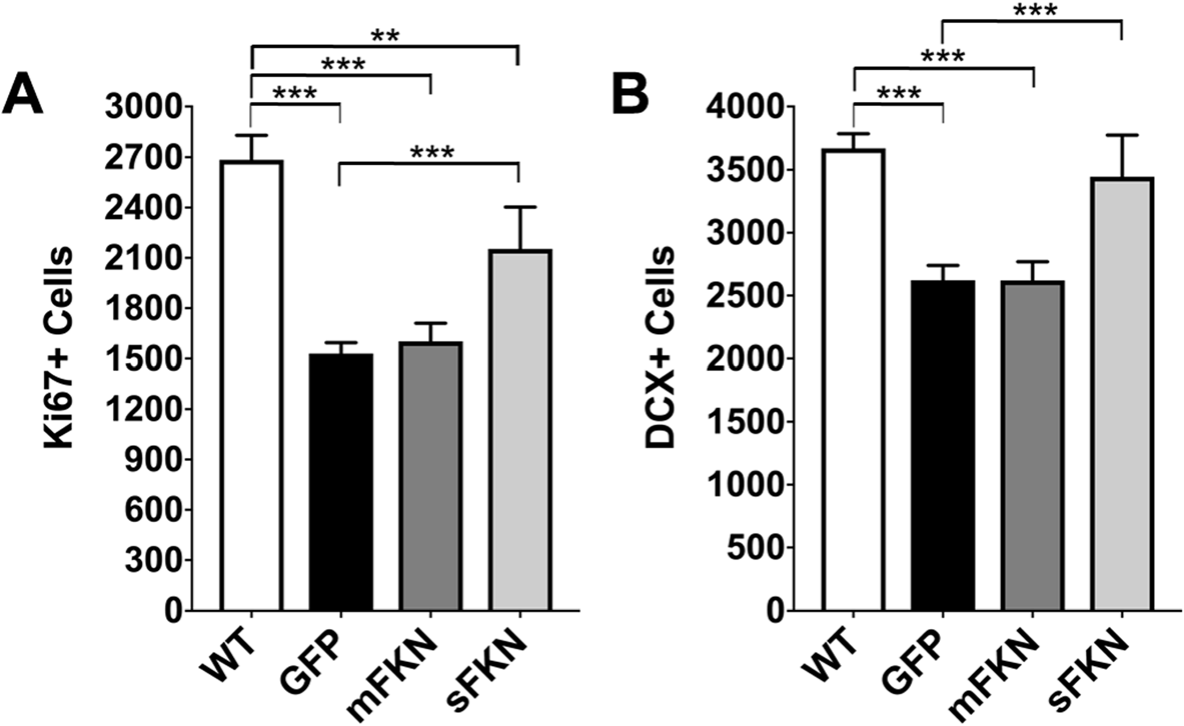
CX3CL1^−/−^ mice show deficits in neurogenesis that are rescued by treatment with sFKN, but not mFKN. **a** Unbiased stereology was used to quantify proliferating cells within the subgranular zone (SGZ) of the dentate gyrus as indicated by staining for Ki67. CX3CL1^−/−^ mice receiving AAV expressing GFP showed a significant decrease in Ki67-postive (Ki67+) cells in comparison to WT controls. This deficit was partially rescued by treatment with sFKN, while treatment with mFKN had no effect. Data were analyzed by one-way ANOVA (*n* = 6, *F* [3, 29] = 26.41, *p* < 0.0001) with Tukey’s test. **b** The number of DCX-positive (DCX+) cells in the SGZ was also quantified by stereology as a marker of neurogenesis. Mice that were administered GFP showed significantly fewer numbers of DCX+ cells in comparison to WT controls. Treatment with mFKN had no effect on this deficit, while treatment with sFKN restored neurogenesis to levels comparable to WT controls. Data were analyzed by one-way ANOVA (*n* = 6, *F* [3, 30] = 20.14, *p* < 0.0001) with Tukey’s test. **p < 0.01 and ***p < 0.001

We have previously shown that CX3CR1 ^−/−^ mice show significant deficits in hippocampal neurogenesis. To evaluate if CX3CL1^−/−^ mice show a similar decrease, we used unbiased stereology to quantify proliferating cells within the SGZ of the DG as indicated by Ki67 staining. CX3CL1^−/−^ mice receiving AAV expressing GFP showed a significant decrease in Ki67-postive (Ki67+) cells in comparison to WT controls. This deficit was partially rescued by treatment with sFKN, while treatment with mFKN had no effect (Fig. 6a). The number of DCX-positive (DCX+) cells in the SGZ was also quantified by stereology as a marker of neurogenesis. Similar to the trend observed with Ki67, mice that were administered GFP showed significantly fewer numbers of DCX+ cells in comparison to WT controls. Treatment with mFKN had no effect on this deficit, while treatment with sFKN restored neurogenesis to levels comparable to WT controls (Fig. 6b).

## Discussion

Our previous work was the first to demonstrate that CX3CR1 plays a physiological role in cognition and memory [13]. In the current study, we further demonstrate that genetic knock out of its ligand, CX3CL1, also produces physiological consequences similar to those observed with receptor knockout. In particular, CX3CL1 knockout significantly impaired hippocampal dependent learning and memory processes in both a fear conditioning task for long-term memory, and a Barnes maze task to assess spatial learning and memory (Figs. 2 and 3). Interestingly, in the Barnes maze, the deficit was a widening of the spatial search pattern during the probe trial, indicating impaired spatial mapping that is correlated with activity in the dentate gyrus [31]. Impaired memory correlated with deficits in hippocampal neurogenesis as demonstrated by a significant decrease in both Ki67+ and DCX+ neurons within the SGZ of CX3CL1^−/−^ animals in comparison to their WT counterparts (Fig. 6). Moreover, recordings from isolated hippocampal slices indicated that CX3CL1^−/−^ mice display a marked deficit in LTP maintenance (Fig. 5). Collectively, these data suggest that cognitive dysfunction in these mice may be the result of impaired synaptic plasticity and reduced neurogenesis. While the phenotype caused by CX3CL1 knockout seems to be somewhat different than that demonstrated by CX3CR1 ^−/−^ mice, these data are in excellent agreement with our previous findings and those of others.

In addition to examining the consequences of CX3CL1 knockout on cognitive function, we also evaluated the ability of both mFKN and sFKN to rescue the effects of CX3CL1 deficiency. Using rAAV to express different forms of the CX3CL1 protein, we determined that sFKN showed a definitive trend towards improving performance in a contextual fear conditioning task for long-term memory while mFKN treatment did not alter the effects of FKN knockout in this test (Fig. 2). This may suggest that sFKN activity is particularly important for executing hippocampal-dependent associative learning and memory tasks. Further, when spatial learning and memory were assessed by Barnes maze, we similarly noted that animals treated with sFKN displayed a significantly altered search pattern when seeking the target hole during the probe trial than their GFP-treated counterparts. Indeed, these mice tended to spend more time in the target zone and significantly less time searching zones adjacent to the target when compared to GFP-treated mice (Fig. 3), signifying that sFKN activity may be broadly important for hippocampal-dependent memory tasks. Additionally, although it did not improve performance in contextual fear conditioning, administration of rAAV expressing mFKN did enhance performance in the Barnes maze in a manner similar to that of sFKN (Fig. 3). This observation could indicate a specific role for mFKN in spatial memory formation, such as an ability to enhance function within the dentate gyrus, independent of neurogenesis.

While both sFKN and mFKN appear to display some activity in enhancing hippocampal-dependent functions in CX3CL1^−/−^ mice, it is noteworthy that only sFKN appears to rescue hippocampal neurogenesis in these animals. Treatment with AAV-sFKN partially restored expression of both Ki67 and DCX in the SGZ, indicative of increased neurogenesis, suggesting that its ability to mitigate cognitive deficits in CX3CL1^−/−^ mice could be dependent on this activity. However, mFKN did not appear to have any effect on neurogenesis in this region despite its ability to improve spatial memory (Fig. 6). In spite of this discrepancy, both sFKN and mFKN appear to partially restore LTP (Fig. 5). While it has been established that neurogenesis can play an important role in facilitating LTP in mice, it has also been observed that mice deficient in hippocampal neurogenesis develop compensatory mechanisms to sustain LTP [32]. Although, mFKN-treated CX3CL1^−/−^ mice showed LTP maintenance, examining a single signal (Fig. 5b) that showed the varied fEPSPs post theta burst indicating inconsistent maintenance of LTP. Collectively, this could suggest that mFKN may play a role in facilitating the formation of such compensatory mechanisms in CX3CL1^−/−^ mice in order to partially restore LTP; however, mFKN signaling may not be sufficient in and of itself to reliably sustain this function.

While our observations on the effect of CX3CL1 knockout and restoration on cognitive function seem to be in good agreement with our prior findings in CX3CR1^−/−^ mice, the effects of CX3CL1 knockout on motor learning and function were different from those previously observed in CX3CR1^−/−^ mice. Indeed, CX3CL1 knockout appeared to enhance motor performance as assessed by a rotarod task in direct contrast to knockout of CX3CR1, which significantly impairs motor function [13]. Despite improved motor performance, however, motor learning, which was measured as the rate at which mice improved in the rotarod task over time, did not show any differences when comparing CX3CL1^−/−^ mice to their WT counterparts (Fig. 4b). Indeed, although CX3CL1^−/−^ animals showed a slight trend towards increased motor learning, this difference did not reach significance when compared to the other treatment groups.

The effects of restoring mFKN and sFKN signaling on rotarod performance were also evaluated in CX3CL1^−/−^ mice. Interestingly, all mice, regardless of treatment, learned the task at a similar rate, indicating no differences in motor learning between groups (Fig. 4b); however, mFKN and sFKN treatment had opposing effects on the overall motor performance. While mFKN-treated mice behaved more similarly to GFP-treated CX3CL1^−/−^ mice, displaying significantly enhanced motor performance and endurance in comparison to WT mice, particularly on day two of the rotarod task, treatment with AAV-sFKN altered motor performance such that it was more similar to that of WT controls (Fig. 4a). Open field observations did not reveal any significant differences in spontaneous locomotor activity between mFKN-treated, sFKN-treated, CX3CL1^−/−^, or WT mice, suggesting that improvements in motor performance were not due to hyperactivity (Fig. 4c). While the process underlying these unexpected results is not clear, it is possible that enhanced motor performance may be due to peripheral effects on tissues outside the CNS such as skeletal muscle or circulating macrophages. Additionally, it is also possible that loss of CX3CL1 activity during development in specific areas of the brain involved in motor function, such as the striatum and cerebellum, may have consequences for overall motor performance. More specifically, CX3CL1 is expressed in high levels within the striatum [33], and loss of this protein may impact the development and maturation of neural pathways associated with motor function. In this context, restoring sFKN signaling may normalize the function of such pathways, while mFKN signaling does not appear to influence this process. In similar fashion, it has recently been observed that CX3CL1 signaling is necessary for activity-dependent synaptic remodeling to occur in the cortex following sensory lesion induced by whisker cutting in mice, and that inhibition of ADAM10, one of the proteases responsible for cleavage of full-length CX3CL1 into sFKN in the brain, impairs this process [30]. This supports the idea that membrane-bound forms of CX3CL1 may not play a significant role in the remodeling of synaptic circuits, a process that could be involved here in normalizing motor function, and suggests instead that this synaptic remodeling may be predominately mediated by sFKN signaling.

Collectively, our data suggest that CX3CL1 signaling plays an important role in maintaining normal cognitive function in mice and demonstrates that a loss of CX3CL1 signaling could underlie the development of cognitive impairment. Moreover, we demonstrate that membrane-bound CX3CL1 and sFKN display differential activities on cognitive function that could affect their suitability as therapeutic targets. As perturbed CX3CL1 signaling has been observed in both aging and disease, the CX3CL1/CX3CR1 axis has garnered significant attention as a potential target for the treatment of several neurodegenerative diseases including Alzheimer’s disease, Parkinson’s disease, ALS, and multiple sclerosis, as well as ischemic stroke [9, 11, 12, 17–25, 29, 34–36]. However, until recently, the importance of considering the differential functions of different forms of the CX3CL1 protein had not been taken into account. With this in mind, our data could have significant implications for the development of treatments targeting the CX3CL1/CX3CR1 axis as it suggests that sFKN has a much greater and more consistent impact on mitigating cognitive deficits than membrane-bound forms of the CX3CL1 protein, and thus may be a better therapeutic candidate for treating diseases with a significant cognitive component. However, there are still significant gaps in our knowledge regarding the functions of these two forms of the CX3CL1 protein as well as limitations to the current study that must be taken into account.

For example, the differences observed between sFKN and mFKN activity in hippocampal-dependent behavioral tasks could be due to differences in overall availability of the two proteins. As CX3CL1 is not expressed by every neuron following rAAV-PhP.B administration, the inability of mFKN to freely diffuse to nearby neurons could significantly impact its activity in comparison to sFKN. Moreover, although equal concentrations of mFKN and sFKN rAAV were administered to the mice, expression of the sFKN protein we observed was approximately 2-fold higher than that observed for mFKN (Fig. 1). Thus, it cannot be ruled out that higher expression levels of sFKN contributed to its greater and more consistent effects on cognitive function, neurogenesis, and LTP in comparison to mice treated with mFKN, which showed expression comparable to WT levels (Fig. 1). This could also suggest that over-expression of sFKN is required to induce positive alterations in cognition in the context of aging or disease; however, further study is needed to test this hypothesis. Furthermore, it is important to note that levels of sFKN detected in WT brain tissue are higher than those observed for membrane-bound forms of the protein, suggesting that sFKN could be more biologically active even at physiological concentrations [9]. As the ELISA used to detect FKN from brain homogenates in this study that detects both forms of the protein present in WT animals, the data presented here for WT mice include the total levels of both sFKN and membrane-bound CX3CL1, with sFKN being the predominate species, while the data for both sFKN- and mFKN-treated animals represent levels of only one form of the protein. Therefore, it is likely that mFKN levels in rAAV-treated animals are also over-expressed in comparison to those of membrane-bound CX3CL1 found in WT animals, though not to the extent of sFKN. With this consideration in mind, it is possible that even over-expression of mFKN may not be sufficient to positively and consistently influence cognition in the context of CX3CL1 depletion. Furthermore, while sFKN shows appeal as a therapeutic agent given its broad range of effects on cognitive processes, several studies to date have highlighted the need to better characterize the effects of sFKN, which contains the entirety of the mucin-like stalk, versus truncated versions of the soluble CX3CL1 ligand comprising only the chemokine domain [37]. These studies have indicated that different versions of the soluble ligand can produce vastly different outcomes in the context of both neuropathic pain and Alzheimer’s disease that is likely linked to their ability to elicit different changes in microglial phenotype [19, 20, 25, 38, 39]. This distinction has likely been a source of significant variation among studies evaluating the potential of the CX3CL1/CX3CR1 axis as a target for therapeutic development and illustrates the need for further study to define the differential roles of all forms of the CX3CL1 protein.

## Conclusions

Our work demonstrates a role for CX3CL1 in both cognitive and motor function and suggests that loss of CX3CL1 signaling is sufficient to induce cognitive impairment that is likely linked to deficits in both hippocampal neurogenesis and LTP. Further, we demonstrate that sFKN and an obligate membrane-bound form of CX3CL1, mFKN, display differential activities in the context of cognitive function. However, while the current study provides evidence that sFKN more consistently and reliably influences cognition than membrane-bound forms of the CX3CL1 protein, and illustrates the therapeutic potential of sFKN as a target for aging and disease, additional considerations are required for future development.

## Data Availability

The datasets used and/or analyzed during the current study are available from the corresponding author on reasonable request.

## Abbreviations

AAV: Adeno-associated virus
ACSF: Artificial cerebrospinal fluid
ADAM: A disintegrin and metalloproteinase
APP: Amyloid precusor protein
CBA: Chicken beta-actin
CX3CL1: Fractalkine
DCX: Doublecortin
DG: Dentate gyrus
fEPSP: Field excitatory post-synaptic potential
GFP: Green fluorescent protein
HA: Hemaglutinin
IL: Interleukin
LPS: Lipopolysaccharide
mFKN: Membrane-bound fractalkine
MPTP: 1-Methyl-4-phenyl-1,2,3,6-tetrahydropyridine
PS1: Presenilin-1
sFKN: Soluble fractalkine
SGZ: Subgranular zone
SOD1: Cu, Zn-superoxide dismutase
TNF: Tumor necrosis factor
vg: Vector genomes
WT: Wild-type
